# Distinctive functional deficiencies in axonal conduction associated with two forms of cerebral white matter injury

**DOI:** 10.1111/cns.13155

**Published:** 2019-05-29

**Authors:** Hong‐Feng Mu, Xu‐Guang Gao, Si‐Cheng Li, Peng‐Ju Wei, Yong‐Fang Zhao, Wen‐Ting Zhang, Yun Wang, Yan‐Qin Gao

**Affiliations:** ^1^ State Key Laboratory of Medical Neurobiology, MOE Frontiers Center for Brain Science, Institutes of Brain Science, and Neurology Department of Zhongshan Hospital Fudan University Shanghai China

**Keywords:** axonal injury, CAPs, corpus callosum, demyelination, white matter stroke

## Abstract

**Aims:**

This study determines whether assessment with compound action potentials (CAPs) can distinguish two different forms of cerebral white matter injury at the functional levels.

**Methods:**

A pure demyelination model was induced in C57/BL6 adult mice by dietary supplementation of cuprizone (0.2%) for 6 weeks. Callosal L‐N5‐(1‐Iminoethyl) ornithine (L‐NIO) hydrochloride (27 mg/mL) was injected into the corpus callosum (CC) to induce a focal white matter stroke (WMS), resulting in both demyelination and axonal injury. White matter integrity was assessed by performing CAP recording, electron microscopy, and immunohistological and luxol fast blue (LFB) staining.

**Results:**

Immunohistological and electron microscopic analyses confirmed the induction of robust demyelination in CC with cuprizone, and mixed demyelination and axonal damage with L‐NIO. Electrophysiologically, cuprizone‐induced demyelination significantly reduced the amplitude of negative peak 1 (N1), but increased the amplitude of negative peak 2 (N2), of the CAPs compared to the sham controls. However, cuprizone did not affect the axonal conduction velocity. In contrast, the amplitude and area of both N1 and N2 along with N1 axonal conduction velocity were dramatically decreased in L‐NIO‐induced WMS.

**Conclusions:**

Concertedly, parameters of the CAPs offer a novel functional assessment strategy for cerebral white matter injury in rodent models.

## 1 INTRODUCTION

White matter (WM) occupies over 40% of the whole brain in an adult human,^1^, ^2^ which underscores its crucial importance to normal brain function^3^, ^4^, ^5^, ^6^, ^7^ and functional recovery after brain injury.^5^, ^8^, ^9^, ^10^, ^11^ While the majority of research on brain injury has until relatively recently focused on injury to gray matter, the importance of characterizing injury to WM is now being appreciated. Indeed, using diffusion tensor imaging, electron microscopy and/or immunohistological techniques, WM damage has been implicated in a myriad of disorders and diseases, such as traumatic brain injury, attention deficit‐hyperactive disorder, autism, Alzheimer's disease, and stroke.^12^, ^13^, ^14^, ^15^, ^16^, ^17^, ^18^ Although these techniques have provided important information about the microstructural changes that occur in WM after injury, less focus has been given to accompanying the functional changes that occur after WM injury.

White matter tissue is composed of both myelinated and unmyelinated axons. Thus, WM injury can be characterized as demyelination and/or axonal damage. As myelinated and unmyelinated axons display different electrophysiological properties,^13^, ^19^ functional studies employing electrophysiological techniques capitalizing on these differences may allow researchers to discern the nature and severity of any underlying WM damage. To this end, the corpus callosum (CC), the largest tract of WM in the brain, has been used extensively in research to study WM function under normal and pathological conditions (including axonal injury and remyelination).^8^, ^20^ The CC contains both myelinated and unmyelinated axons; thus, it is an ideal structure in which to characterize the electrophysiological response of WM in response to injury or disease.

Compound action potentials (CAPs) have been widely used to investigate the functional alteration of nerve fibers caused by brain injury or WM degeneration.^21^, ^22^, ^23^ Based on previous research, there are typically two types of phase peaks in CAPs traces.^9^, ^13^, ^24^ The early peak (N1) signifies the conduction of myelinated axons, while the later phase peak (N2) represents unmyelinated axons. Previous research has observed heterogeneity in the CAPs response within the rostral‐caudal extent of the CC that appears to be linked to variations in the proportion of myelinated and unmyelinated axons.^19^ We therefore surmised that demyelination and axonal damage might give different CAPs profiles relative to the normal CAPs response. In the present study, we aimed to test this hypothesis by performing a comprehensive comparison of the CAPs response in the CC between demyelination induced by cuprizone^25^, ^26^ and white matter stroke (WMS),^27^ which causes both demyelination and axonal damage. Evidence garnered from electrophysiological experiments was substantiated with EM and immunohistological analysis.

We found that the N1 and N2 components of the CAPs undergo distinct alterations under conditions of demyelination and demyelination combined with axonal injury. The CAPs’ amplitude and area cannot solely distinguish these two different disease models. Other parameters of the CAPs, such as the half‐width and conduction velocity, need to be considered in concert with the amplitude and area to make inferences regarding the nature of the underlying WM damage.

## 2 METHODS

### 2.1 Animal

Adult male C57BL/6J mice (8‐12 weeks old) were purchased from the Chinese Academy of Sciences (Shanghai). Mice were housed in a temperature‐ and humidity‐controlled animal facility with a 12‐hour light/dark cycle and freely available food and water. All animal experiments were approved by Fudan University's Institutional Animal Care and Use Committee and performed in accordance with the Chinese National Science Foundation animal research regulations and National Institutes of Health's Guide for the Care and Use of Laboratory Animals.

### 2.2 Induction of experimental demyelination

Mice were subjected to demyelination as previously described.^25^ Specifically, mice were fed a diet containing 0.2% cuprizone (Sigma‐Aldrich) mixed with their standard laboratory rodent diet (Teklad Global 2918; Harlan) for 6 weeks. The sham mice were fed with standard laboratory rodent chew without cuprizone supplementation. Cuprizone administration at this dose leads to selective apoptosis of oligodendrocytes, which subsequently results in demyelination.^25^

### 2.3 Induction of white matter stroke

Mice were randomly divided into Sham or L‐NIO groups, and WMS was induced as previously described 27 (Appendix S1).

### 2.4 CAP measurements

Compound action potentials in the CC were evaluated as previously described^28^ (Appendix S2).

### 2.5 Tissue section preparation

Mice were anesthetized with 3% isoflurane in 67:30% N_2_O/O_2_ until they were unresponsive to tail pinch, and transcardially perfused with saline followed by 4% paraformaldehyde in 0.1 mol/L phosphate buffer (pH 7.4). Brains were removed and transferred to a 30% sucrose solution in phosphate‐buffered saline overnight for cryoprotection. Serial coronal brain sections were sliced at 25 µm on a cryostat (CM1900; Leica) beginning 1.1 mm anterior to bregma.

### 2.6 Immunohistochemical staining

Brain sections were immunohistochemically stained for MBP, SMI32, NF200, and densitometric analysis was performed as previously described^28^ (Appendix S3).

### 2.7 Luxol fast blue staining

Luxol fast blue (LFB) was used to stain for damaged myelin in the CC performed as previously described.^29^ The 25‐μm‐thick coronal sections were incubated in the 0.1% LFB stain (Cat.S3382; Sigma) overnight, at 60°C. Then, slides were rinsed in 95% ethanol followed by double‐distilled water (ddH_2_O), and differentiated with 0.05% lithium carbonate. Slides were then rinsed in 70% ethanol and ddH_2_O. The differentiation step was repeated until there was a sharp contrast between the CC and the cortex.

### 2.8 Electron microscopic studies

Electron microscopic analysis was used to assess axonal morphology/damage in the CC, as described previously^28^ (Appendix S4).

### 2.9 Statistical analysis

Statistical analyses were performed using GraphPad Prism 7.0 (GraphPad Software). Data are expressed as the mean ± standard error of mean (SEM) and were analyzed by analysis of variance (ANOVA) followed by post hoc Bonferroni/Dunn tests. The difference in means between two groups was assessed by the two‐tailed Student *t*‐test. Differences among multiple groups were analyzed with one‐way ANOVA *P* < 0.05 was considered statistically significant.

## 3 RESULTS

### 3.1 Different CAP responses between two coronal levels of brain slices

**Figure 1:**
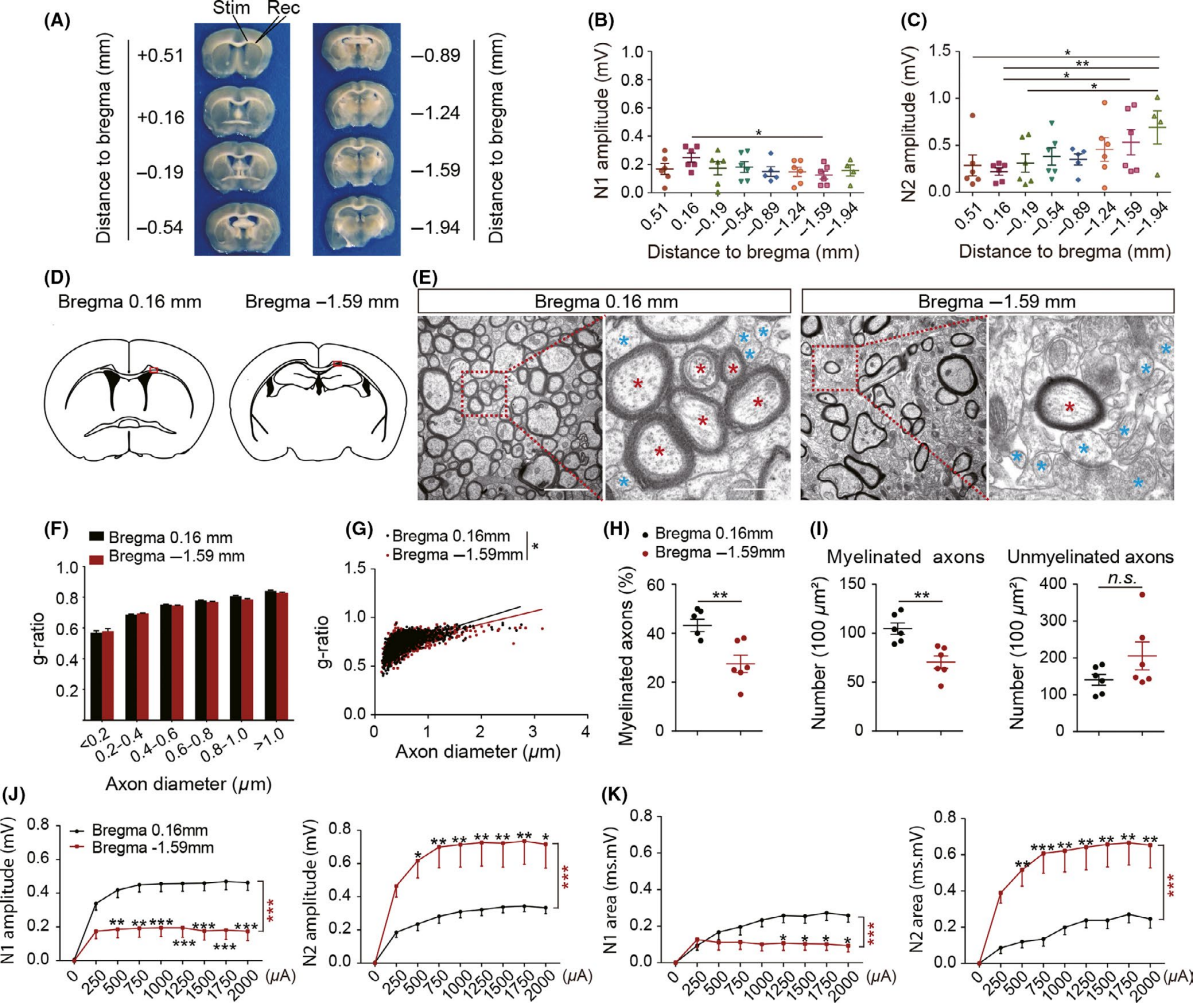
Compound action potentials (CAPs) responses were related to myelination as verified by electron microscopy. A, Representative 350‐μm‐thick coronal brain sections at different distances from bregma used for CAPs recording. Stimulating and recording electrodes were positioned at the CC as shown to measure the evoked CAPs. B, Signal conduction along myelinated axons, as measured by the amplitude of the N1 component of the CAPs in response to a 2 mA stimulus in different sections from the mouse brain. n = 4‐6 mice. C, Signal conduction along unmyelinated axons, as measured by the amplitude of the N2 component of the CAPs in response to a 2 mA stimulus in different sections from the mouse brain. n = 4‐6 mice. D, Illustration of brain sections at bregma + 0.16 mm and bregma − 1.59 mm. Red boxes indicate regions subjected to electron microscopy. E, Representative electron micrographs from brain sections at bregma + 0.16 mm and bregma − 1.59 mm. The red asterisks indicate myelinated axons, and blue asterisks indicate unmyelinated axons. Scale bar = 2 μm (left) and 0.5 μm (right). F, Comparison of g‐ratio as a function of axon diameter in brain sections at bregma + 0.16 mm and bregma − 1.59 mm. G, Scatter plot of g‐ratio as a function of total axon diameter in the two brain sections. H, The percentage of myelinated axons in electron micrographs calculated for brain sections at bregma + 0.16 mm and bregma − 1.59 mm. Quantification of myelinated axons and myelin thickness was done in five representative images/animals. I, Quantification of myelinated (left panel) and unmyelinated axons (right panel) in electron micrographs from brain sections at bregma + 0.16 mm and bregma − 1.59 mm. Quantification of myelinated and unmyelinated axons was done in five representative images/animals. J, Comparison of N1 (left panel) and N2 (right panel) amplitudes between brain sections at bregma + 0.16 mm and bregma − 1.59 mm under different stimulus strength (0.25‐2 mA). K, Comparison of N1 (left panel) and N2 (right panel) area between brain sections at bregma + 0.16 mm and bregma − 1.59 mm under different stimulus strength (0.25‐2 mA). The results are presented as the mean ± SEM. *P < 0.05, **P < 0.01, ***P < 0.001; ns, statistically non‐significant difference. n = 6‐10 mice

Measurement of CAPs has been shown to be a key way to assess the functional integrity of axons.^22^, ^30^ However, we have noticed that the amplitude of CAPs varies with the brain slice being tested and hypothesize that this variability derives from inherent differences in the proportion of myelinated to unmyelinated axons in brain slices from different locations. Therefore, in this study, CAPs were recorded in 8 consecutive 350‐µm‐thick brain coronal slices spanning from distance to bregma 0.51 to −2.29 mm (Figure 1A). Additionally, we observed that N1 of the CAPs was obscured by a stimulus artifact if the incubating temperature was close to physiological temperatures (35‐37°C), or the distance between the stimulus electrode and the recording electrode was too short even at room temperature. As a result, all experiments were performed in a CSF at room temperature (22°C) with a 0.75 mm distance between electrodes.

The N1 amplitude showed an overall downward trend from more anterior to posterior slices. However, the only statistically significant difference was between the slice taken +0.16 mm from bregma (0.25 ± 0.08 mV, n = 6) and the slice taken −1.59 mm from bregma (0.13 ± 0.03 mV, n = 6, *P* < 0.05; Figure 1B). In contrast, N2 amplitudes mainly increased from anterior to more posterior slices in the mouse brain. Specifically, the N2 amplitude at bregma −1.59 mm (0.53 ± 0.13 mV, n = 6, *P* < 0.05) and at bregma −1.94 mm (0.69 ± 0.17 mV, n = 4, *P* < 0.01) was over two times higher than that at bregma + 0.16 mm (0.22 ± 0.04 mV, n = 6; Figure 1C). And N2 amplitudes at bregma + 0.51 mm (0.29 ± 0.17 mV, n = 6, *P* < 0.05) and −0.19 mm (0.31 ± 0.17 mV, n = 6, *P* < 0.05) were also significantly lower than that at bregma −1.94 mm. These results suggest that the distribution of myelinated and unmyelinated axons may differ between the anterior and posterior aspects of the mouse brain.

### 3.2 Correlations between CAP responses and axonal myelination

Based on the electrophysiology data from the anterior to posterior coronal brain sections, we hypothesized that there were more myelinated axons in anterior portions than in posterior portions of the mouse brain. To test this hypothesis, we performed electron microscopy (EM) to compare the microstructure in the CC from two brain sections that differed mostly in their N1 and N2 components of the CAPs (bregma +0.16 mm vs bregma −1.59 mm, Figure 1D,E). Firstly, we assessed the g‐ratio of myelinated axons, which reflects myelin thickness, in the brain slices from these two positions. Although there were no significant differences in the g‐ratio across different axon diameters between the anterior and posterior coronal slices (Figure 1F), there was a significant difference in g‐ratio as a function of overall axon diameter (Figure 1G).

We quantified 100‐210 and 100‐300 axons/animal in coronal sections at the levels of bregma +0.16 and −1.59 mm, respectively. Both the proportion (43.2 ± 2.6% and 27.5 ± 3.6%, *P* < 0.01; Figure 1H) and the number (104.7 ± 5.8 per 100 μm^2^ and 70.5 ± 6.4 per 100 μm^2^, *P* < 0.01; Figure 1I) of myelinated fibers were significantly higher in the bregma +0.16 mm sections than those in the bregma −1.59 mm sections. In contrast, there was no discernible difference in the number of unmyelinated fibers between the bregma +0.16 mm (62.8 ± 2.8 per 100 µm^2^) and −1.59 mm section (70.3 ± 2.9 per 100 µm^2^; Figure 1I).

**Figure 2:**
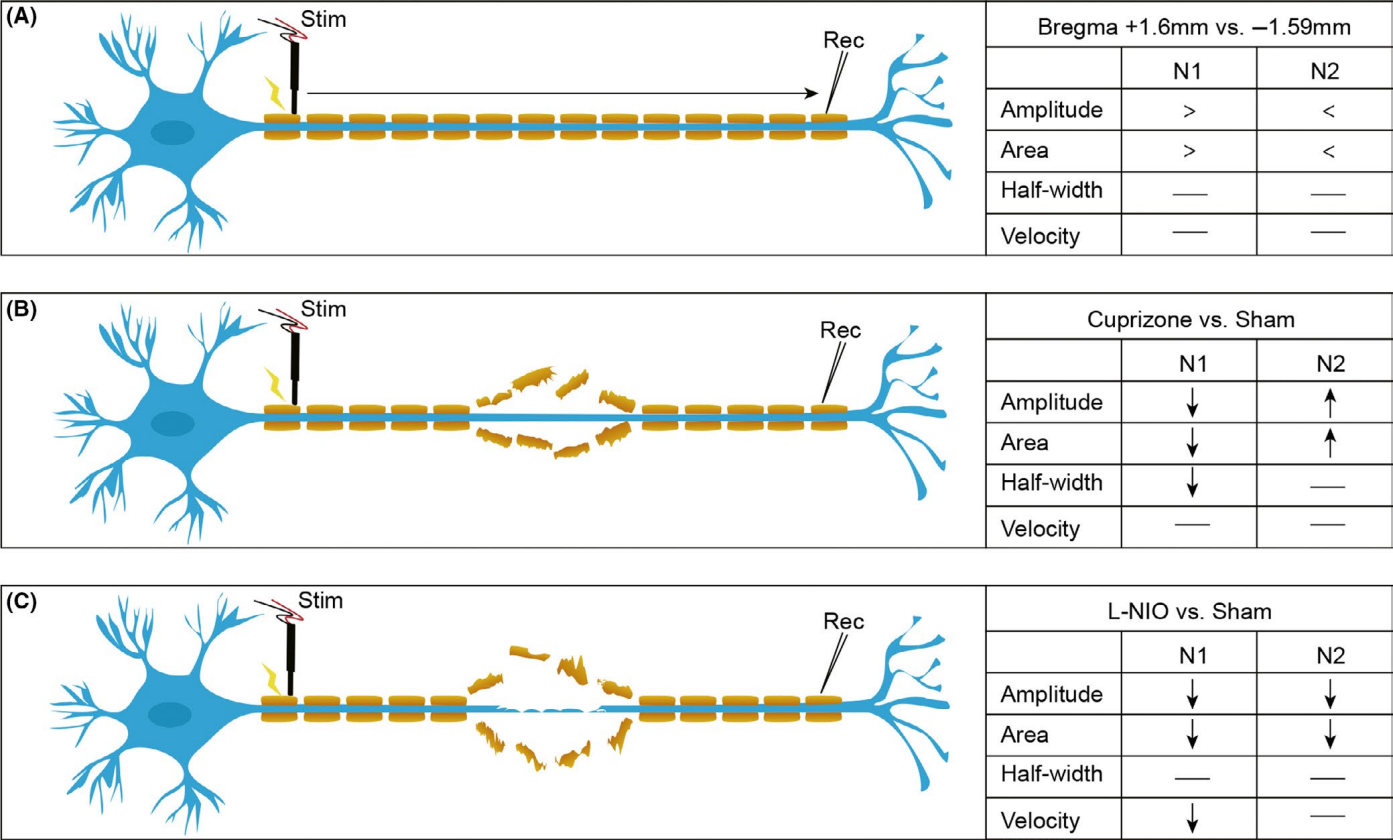
Comparisons of CAPs responses among sham, cuprizone‐induced demyelination, and WMS mice. A, Axonal conduction diagram under physiological conditions. The amplitudes and area could distinguish different degrees of myelinated areas under physiological conditions. B, Axonal conduction diagram under cuprizone‐induced pure demyelination conditions. Demyelination could be distinguished by amplitudes, area, and half‐width. C, Axonal conduction diagram under L‐NIO‐induced mixed demyelination and axonal damage conditions. The axonal injury involved WM injury could be distinguished by amplitudes, area, and velocity

These results indicated that the number of myelinated axons predominates in anterior aspects of the mouse brain. In accordance, we observed a significantly higher N1 amplitude and area as functional responses to the stimulus in more anterior slices (bregma +0.16), as opposed to the more posterior slices (bregma −1.59), where the N2 amplitude and area were significantly higher (*P* < 0.05‐0.001; Figure 1J,K). However, there were no significant differences in conduction velocity and half‐width of CAPs between brain slices at bregma +0.16 and −1.59 mm (Figure S1). The healthy myelinated axons under physiological conditions are composed of intact axons wrapped with myelin sheath (Figure 2A).

### 3.3 Deficiencies in CAP responses in the model of cuprizone‐induced demyelination

**Figure 3:**
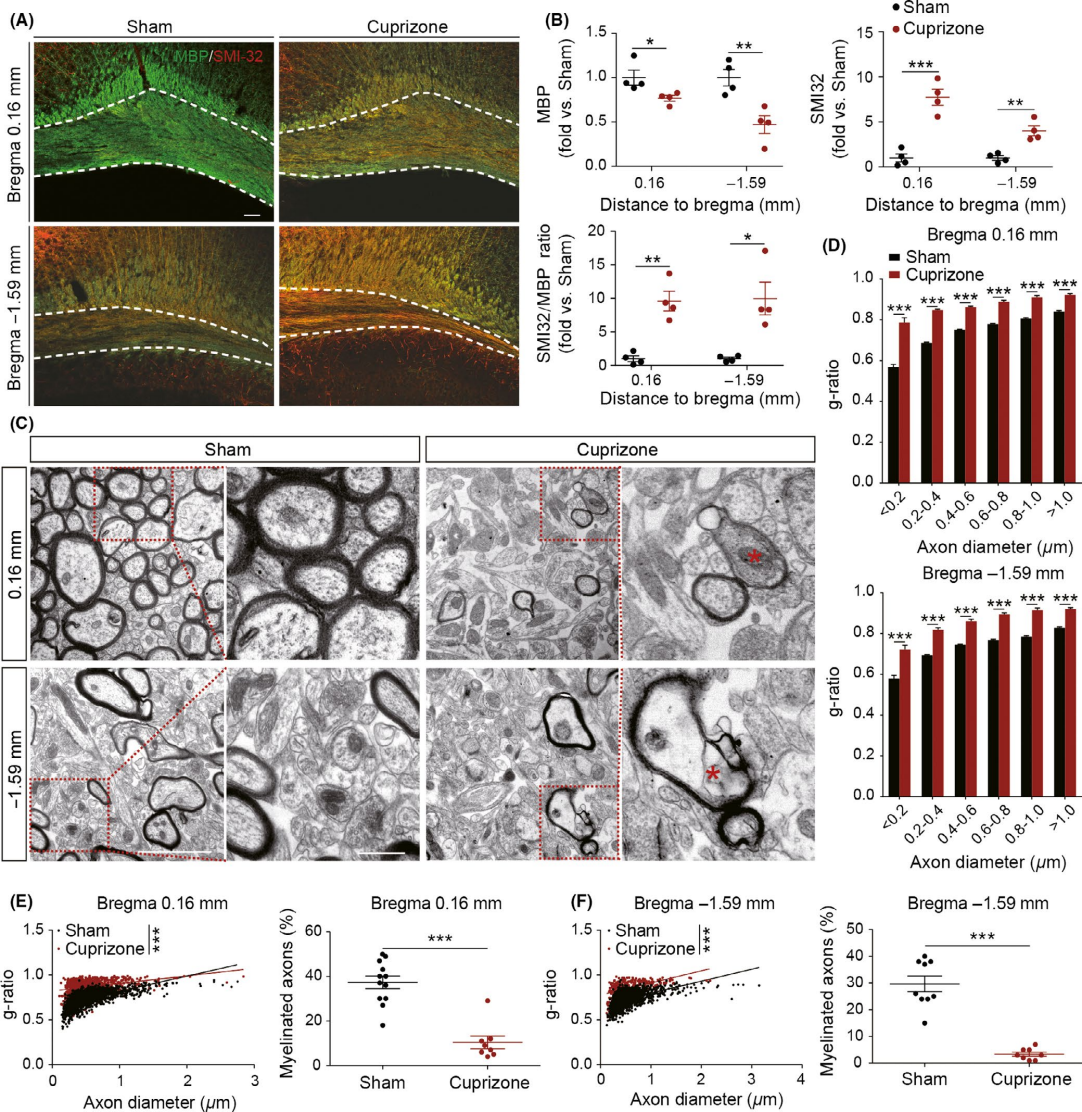
Demyelinating process of the corpus callosum in cuprizone‐induced mice. A, Representative images of MBP/SMI‐32 double immunofluorescent staining in the corpus callosum (CC) on brain sections at bregma + 0.16 mm and bregma − 1.59 mm after 6 wk cuprizone diet. The area between white dashed lines was quantified. Scale bar = 100 μm. B, Quantification of t SMI‐32 (above panel) and MBP (below panel) fluorescence intensity in CC (n = 4 mice/group). C, Representative electron micrographs from brain sections at bregma + 0.16 mm and bregma − 1.59 mm from normal and 6 wk cuprizone diet‐induced demyelinating mice. The red asterisks indicate demyelinating axons. Scale bar = 2 μm (left) and 0.5 μm (right). D, Comparison of myelination thickness as a function of axon diameter on brain sections at bregma + 0.16 mm (left panel) and bregma − 1.59 mm (right panel) between normal and demyelinated mice. E, Scatter plot of g‐ratio distribution as a function of axon diameter in normal and demyelinated axons (left panel) and percentage of myelinated axons in normal and demyelinated mice (right panel) on brain sections at bregma + 0.16 mm. F, Scatter plot of g‐ratio distribution according to different scales of axon diameter in normal and demyelinated axons (left panel) and percentage of myelinated axons in normal and demyelinated mice (right panel) on brain sections at bregma − 1.59 mm. All data are presented as the mean ± SEM. *P < 0.05, **P < 0.01, ***P < 0.001. n = 6‐8 mice/group

Previous studies have demonstrated that chronic ingestion of cuprizone results in significant demyelination of the CC,^31^, ^32^, ^33^ along with demyelination in the hippocampus and cortical layers. Therefore, to better characterize what the degree of demyelination alters the electrical properties of axons, we compared CAPs in the anterior and posterior CC in mice maintained on a normal diet or cuprizone diet for 6 weeks. To verify demyelination of axons after feeding mice with cuprizone, we labeled brain slices with MBP and SMI‐32 immunofluorescence. As expected, MBP immunofluorescence in the CC in cuprizone diet mice was significantly decreased to 0.77 ± 0.09 (*P* < 0.05) and 0.47 ± 0.14 (*P* < 0.01) of the sham control normal diet mice, at the coronal levels of bregma +0.16 mm and −1.59 mm, respectively (Figure 3A,B), indicative of cuprizone‐induced myelin damage. Maintenance on the cuprizone diet also induced significant increases in SMI‐32 immunofluorescence by 7.73 ± 0.99 (*P* < 0.001) and 3.99 ± 0.62 (*P* < 0.01)‐folds at bregma +0.16 mm and −1.59 mm coronal levels, respectively (Figure 3A,B). The above changes in SMI‐32 and MBP immunofluorescence resulted in remarkable increases in the SMI‐32/MBP ratio in cuprizone‐treated mice compared to controls under normal diet (Figure 3B).

Next, we performed electron microscopy to verify microstructural changes in the CC of mice fed with the cuprizone diet (Figure 3C). The g‐ratio was significantly increased in cuprizone‐fed mice at both bregma +0.16 mm and bregma −1.59 mm in accordance with a greater proportion of demyelinated axons in these sections (Figure 3D‐F).

**Figure 4:**
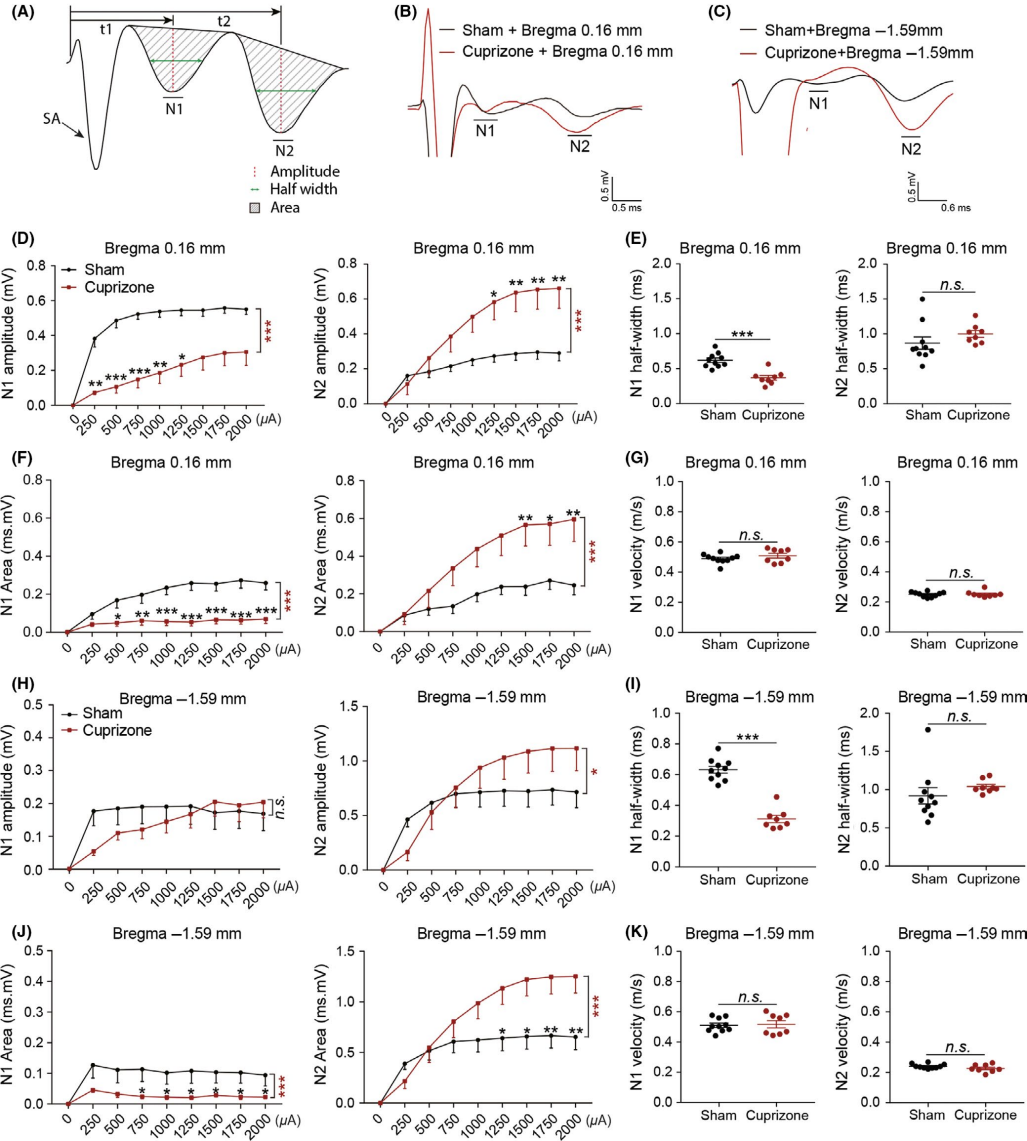
Demyelination leads to electrical property changes in normal and demyelinated mice. A, Illustration of N1 and N2 locations and measured parameters on a compound action potential (CAP) trace. The black arrow indicates stimulus artifact (SA). t1 and t2 indicate conduction time of N1 and N2, respectively. The conduction velocity = conduction distance (0.75 mm)/conduction time (t1 or t2). B, Representative traces of the evoked CAPs in the CC (stimulus, 2 mA) on brain sections from normal and demyelinated mice at bregma + 0.16 mm. C, Representative traces of the evoked CAPs in the CC (stimulus, 2 mA) on brain sections from normal and demyelinated mice at bregma − 1.59 mm. D, Comparison of N1 (left panel) and N2 (right panel) amplitudes under different stimulus strength (0.25 − 2 mA) bregma + 0.16 mm between normal and demyelinated mice. E, Comparison of N1 and N2 half‐width between brain sections at bregma + 0.16 mm between normal and demyelinated mice. F, Comparison of N1 (left panel) and N2 (right panel) area under different stimulus strength (0.25‐2 mA) at bregma + 0.16 mm between normal and demyelinated mice. G, Comparison of N1 and N2 conduction velocity between brain sections at bregma + 0.16 mm between normal and demyelinated mice. H, Comparison of N1 (left panel) and N2 (right panel) amplitudes under different stimulus strength (0.25‐2 mA) at bregma − 1.59 mm between normal and demyelinated mice. I, Comparison of N1 and N2 half‐width between brain sections at bregma − 1.59 mm between normal and demyelinated mice. J, Comparison of N1 (left panel) and N2 (right panel) area under different stimulus strength (0.25‐2 mA) at bregma − 1.59 mm between normal and demyelinated mice. K, Comparison of N1 and N2 conduction velocity between brain sections at bregma − 1.59 mm between normal and demyelinated mice. All data are presented as the mean ± SEM. *P < 0.05, **P < 0.01, ***P < 0.001; ns, statistically non‐significant difference. n = 8‐10 mice/group

Finally, our electrophysiological CAP measurements in the CC showed that the N1 amplitude was significantly decreased at bregma +0.16 mm in cuprizone‐fed mice (*P* < 0.001; Figure 4D) compared to normal diet‐fed mice. This decrease in the N1 amplitude in cuprizone‐fed mice corresponded with significant decrease in the N1 area (*P* < 0.001; Figure 4F). In contrast, the mean N2 amplitude was significantly higher in the cuprizone‐fed mice compared to normal fed mice (*P* < 0.01; Figure 4D), which corresponded with an increase in the N2 area (*P* < 0.001; Figure 4F). The N1 amplitudes were not significantly different in the more posterior brain slice at bregma −1.59 mm between the normal diet and cuprizone‐fed mice, whereas the N2 amplitude and area were increased as a function of total stimulating strengths in the cuprizone‐fed mice (Figure 4H). Although demyelination induced by cuprizone did not markedly alter the N1 and N2 amplitude, it significantly decreased the N1 area and increased the N2 area at bregma −1.59 mm (Figure 4J). Further, the conduction velocity for N1 and N2 was not altered by cuprizone in either the anterior or posterior slice (Figure 4G,K), but cuprizone suppressed the half‐width of the N1 component (calculations based on stimulus intensity of 1 mA) of the CAPs in both the anterior and posterior slices (*P* < 0.001; Figure 4E,I), without any effects on the half‐width of N2 (calculations based on stimulus intensity of 2 mA). Our results demonstrated that myelinated fibers lost myelin sheath without axonal injury under six‐week cuprizone dietary supplementation (Figure 2B). Our data suggested that measurement of the CAPs using parameters, such as amplitudes, areas, or half‐width, could detect axonal demyelination at functional levels (Figure 2B).

### 3.4 Deficiencies in CAP responses in the model of WMS

**Figure 5:**
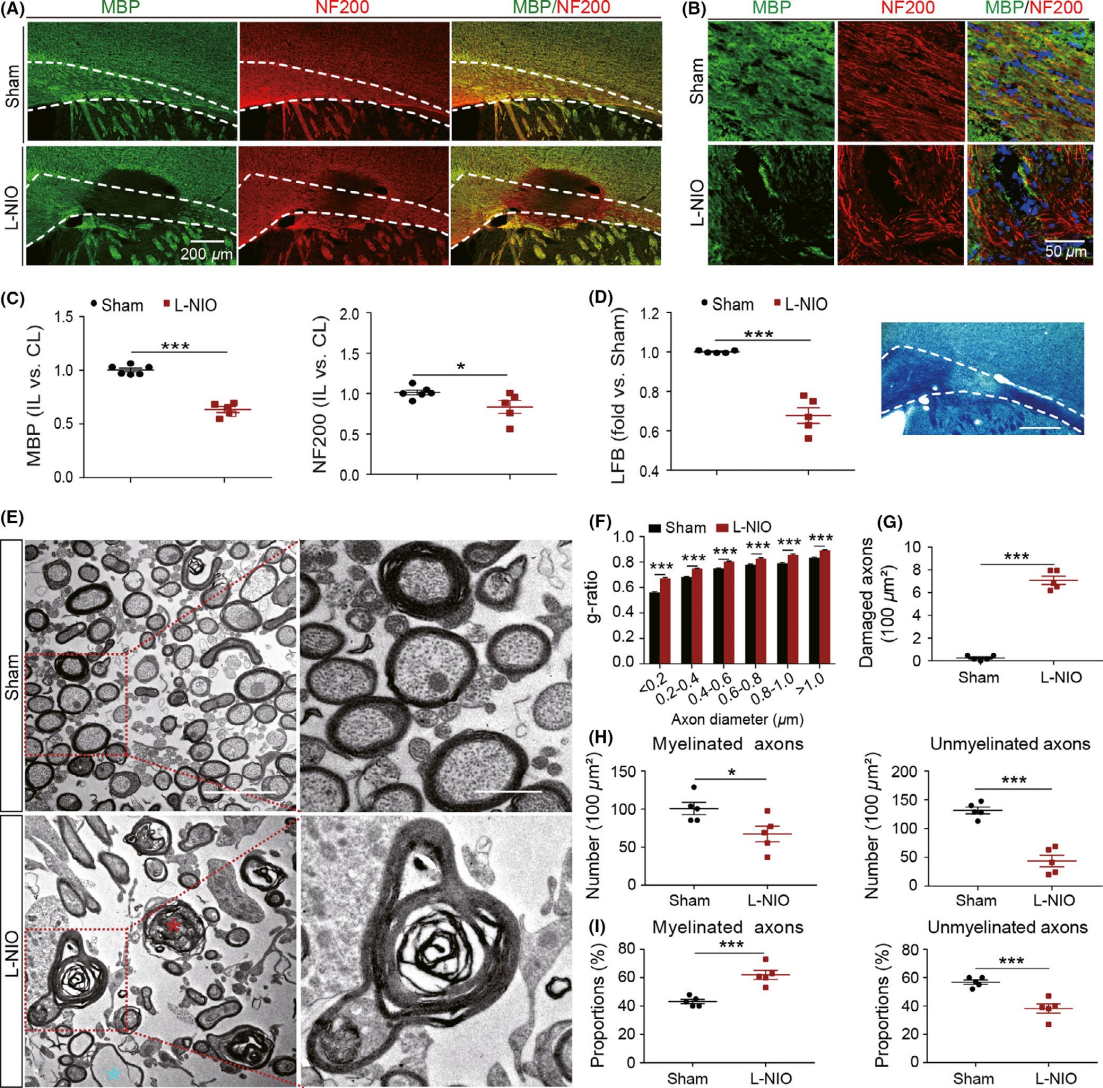
Demyelination and axonal injury of the corpus callosum coexist in L‐NIO‐induced WMS mice. A, Representative images of double immunofluorescent staining for MBP (green) and NF200 (red) in the CC on brain sections at bregma + 0.16 mm at 7 d after WMS. The area between white dashed lines was quantified. Scale bar = 200 μm. B, Representative high power images of lesion. Scale bar = 50 μm. C, Quantification of MBP lesion and NF200 fluorescence intensity in the corpus callosum (CC). D, Representative staining image of LFB (right panel) and quantification of LFB intensity (left panel) at 7 d after WMS. Scale bar = 200 μm. E, Representative electron micrographs from brain sections at bregma + 0.16 mm from normal saline (NS) and L‐NIO groups at 7 d after WMS. Red asterisks indicate injured axons, and blue asterisks indicate myelin without axons. Scale bar = 2 μm (left) and 0.5 μm (right). F, Comparison of myelination thickness based on different scale of axon diameter on brain sections at bregma + 0.16 mm between NS and L‐NIO groups at 7 d after WMS. G, Quantification of damaged axons in electron micrographs from brain sections at bregma + 0.16 mm between normal and WMS mice. Quantification of damaged axons was done in five representative images/animals. H, Quantification of myelinated (left panel) and unmyelinated axons (right panel) in electron micrographs from brain sections at bregma + 0.16 mm between normal and WMS mice. Quantification of myelinated and unmyelinated axons was done in 5 representative images/animals. I, Compare the percentages of myelinated (left panel) and unmyelinated axons (right panel) from brain sections at bregma 0.16 mm between NS and L‐NIO groups at 7 d after WMS. (n = 5 mice/group. All data are presented as the mean ± SEM. *P < 0.05, ***P < 0.001. n = 5‐6 mice/group

Demyelination with cuprizone was characterized by alterations in amplitude, area, and half‐width of the N1 and/or N2 component of the CAPs. To ascertain what influence a more complex injury would have on the electrical properties of CC axons, L‐NIO was used to induce axonal injury concurrent with demyelination. The brains were harvested at 7 days after L‐NIO or saline injection. Demyelination and axonal injury were verified by immunostaining with MBP and NF200 (Figure 5A,5), respectively, as well as by LFB staining (Figure 5D) at 7 days after L‐NIO infusion. In mice subjected to L‐NIO‐induced WMS, the immunofluorescence intensity in the CC lesion was significantly decreased to 0.64 ± 0.03 (*P* < 0.001) for MBP and 0.83 ± 0.08 (*P* < 0.05) for NF200 staining of the contralateral CC (Figure 5C). A significant decrease in LFB staining further confirmed these results (Figure 5D, *P* < 0.001).

Electron micrographs (Figure 5E) show that L‐NIO induced both demyelination and axonal damage, as indicated by dramatically increased g‐ratio (Figure 5F, *P* < 0.001), significantly increased numbers of damaged axons (Figure 5G, *P* < 0.001), and significantly decreased numbers of both myelinated (Figure 5H, *P* < 0.05) and unmyelinated axons (Figure 5H, *P* < 0.001). The damaged axons contained enormous empty vacuoles and unevenly distributed axoplasm. Although the numbers of myelinated and unmyelinated axons were both decreased, the proportions of remaining myelinated axons were significantly increased (Figure 5I, *P* < 0.001), while the proportions of remaining unmyelinated axons were decreased significantly (Figure 5I, *P* < 0.001). These quantitative data on axon numbers suggested that while both myelinated and unmyelinated axons were damaged by L‐NIO, the unmyelinated axons might represent the more vulnerable target for L‐NIO‐induced WMS.

**Figure 6:**
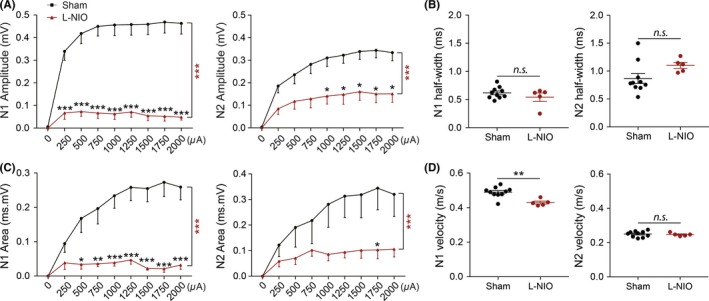
Combined demyelination and axonal injury contribute to corpus callosum property changes in L‐NIO‐induced WMS mice. A, Comparison of N1 (left panel) and N2 (right panel) amplitudes under different stimulus strength (0.25‐2 mA) on brain sections at bregma + 0.16 mm between NS and L‐NIO groups at 7 d after WMS. B, Comparison of N1 and N2 half‐width between brain sections at bregma + 0.16 mm between NS and L‐NIO groups at 7 d after WMS. C, Comparison of N1 (left panel) and N2 (right panel) area under different stimulus strength (0.25‐2 mA) in those two selected brain sections at bregma + 0.16 mm between NS and L‐NIO groups at 7 d after WMS. D, Comparison of N1 and N2 conduction velocity between brain sections at bregma + 0.16 mm between NS and L‐NIO groups at 7 d after WMS. n = 5‐10 mice/group. All data are presented as the mean ± SEM. *P < 0.05, **P < 0.01, ***P < 0.001; ns, statistically non‐significant difference. n = 5‐10 mice/group [Colour figure can be viewed at wileyonlinelibrary.com]

L‐NIO produced suppression of the N1 and N2 components of the CAPs, as indicated by a downward shift in both amplitude and area of these two components (Figure 6A,6). There was also a significant decrease in the N1 conduction velocity (*P* < 0.01), whereas the N2 conduction velocity and half‐widths of both N1 and N2 components did not show any significant change (Figure 6B,6). These data indicated that L‐NIO‐induced WMS exhibits functional deficiencies in CAP responses with the suppression of both N1/N2 amplitudes and N1 conduction velocity. These CAP changes (reduction in both amplitudes and conduction velocity) in response to L‐NIO injection were consistent with the histopathological observations in this WMS model, in which both myelin loss and axonal injury occurred (Figure 2C). Thus, the mixed WM injury involving both demyelination and axonal injury can be distinguished at the functional levels from the pure demyelination model induced by cuprizone, as the latter does not cause the suppression of conduction velocities.

## 4 DISCUSSION

Disruption in WM maturation leads to abnormal brain function correlated with numerous neuropsychological disorders.^16^, ^34^, ^35^, ^36^ Furthermore, WM injury occurs in various CNS conditions/diseases, such as attention deficit‐hyperactive disorder, autism, multiple sclerosis, Alzheimer's disease, TBI, and stroke.^12^, ^13^, ^14^, ^15^, ^16^, ^17^, ^37^ WM recovery is also related to improved neurological function.^38^, ^39^, ^40^, ^41^ Therefore, maintaining the functional integrity of WM tracts is essential to normal brain development, function, and recovery. Moreover, understanding how WM tracts are altered functionally in response to injury may provide crucial insight into the underlying structural damage to WM.

The present study examined whether functional changes in the electrophysiological properties of axons in response to two forms of WM injury could indeed provide insight into the nature of the underlying WM damage. Parameters of the N1 (myelinated axons) and N2 (unmyelinated axons) components of the CAPs were contrasted in the anterior CC after cuprizone and L‐NIO induced WM injury mouse models. Brain slices from the anterior CC were chosen for this comparative analysis, similar as a previous study,^19^ our data presented in the current study have showed that the anterior CC contains a higher proportion of myelinated axons than the posterior CC. The results indicated that the parameters beyond the amplitude and the area of the N1 component of the CAPs might be used to distinguish between either pure demyelination or demyelination concurrent with axonal injury. Indeed, there is widespread consensus that demyelination and axonal injury both suppress the amplitude and area of the myelinated component of the CAP,^42^, ^43^ as observed in the present study. The decrease in these parameters signifies a decrease in the number of myelinated axons participating in the CAP.^19^ In some cases, spontaneous or therapy‐induced remyelination attenuated this decrease,^42^, ^44^ which provides direct evidence that under some conditions demyelination per se caused the decrease in both the amplitude and area of the N1 component of the CAP. A time course analysis of the CAP was not conducted in the current study to ascertain whether remyelination would reverse the decrease in the N1 component, but such behavior has been previously observed after cuprizone‐induced demyelination.^44^

We have, however, observed an increase in both the amplitude and area of the N2 (unmyelinated) component of the CAP, as might be expected in a pure demyelination model, as previously myelinated axons shifted to a more unmyelinated profile. This decrease in myelinated axons concomitant with an increase in unmyelinated axons may serve as a functional proof that demyelination was the predominant form of WM damage in the current study with cuprizone. Indeed, immunohistological and EM analyses substantiated the pure demyelination nature of our cuprizone model. The rostrocaudal differences in corpus callosum demyelination have been reported previously using magnetic resonance imaging after six weeks of cuprizone induction.^45^ In general, cuprizone‐induced demyelination is more severe in the genu of the corpus callosum,^45^ and our CAPs results are consistent with this pattern. In particular, we found that the N1 amplitudes were significantly decreased in brain sections at the level of bregma 0.16 mm, but not at the level of bregma −1.59 mm (Figure 4). The decrease in the number of myelinated axons induced by cuprizone was also associated with a decrease in the temporal distribution of the CAP leading to a more synchronous response, as suggested by the decrease in the CAP half‐width.^19^ This suggests greater homogeneity in the axons recruited for the N1 component of the CAP. Although cuprizone and L‐NIO both reduced the number of myelinated axons recruited for the CAP, differences between the two WM injury models with respect to other parameters of the CAP point to dissimilarities in the underlying cause. Whereas cuprizone increased the number of unmyelinated axons participating in the N2 component of the CAP, as previously discussed, L‐NIO reduced the number of unmyelinated axons recruited to the N2 component. These data suggest that unlike with cuprizone, a proportion of demyelinated axons in the L‐NIO model may have quickly begun to degenerate after injury. The decrease in NF200 immunostaining supports this contention. Functional evidence of potential structural damage to myelinated axons was a decrease in their conduction velocity, as this parameter is directly correlated with axon diameter and myelination.^46^ In the cuprizone model, our result showed that demyelination alone did not have any effects on the conduction velocity. Previous studies have observed a compensatory increase in mitochondrial activity in demyelinated axons that may function to maintain the conduction velocity.^47^, ^48^, ^49^ Although we did not assess mitochondria in the current study, such an increase could explain the lack of effect of pure demyelination alone on the conduction velocity. Immunohistologically, loss of MBP and LFB staining verified demyelination one week after L‐NIO infusion. Quantitative analysis of electron micrographs at this timepoint also showed demyelinated axons, along with axonal injury, and a decrease in the number of myelinated and unmyelinated axons, consistent with the functional deficits observed in the CAP.

## 5 CONCLUSION

In conclusion, under normal physiological and pathological conditions, myelinated and unmyelinated axons display distinct patterns of electrophysiological behavior. Under pathological conditions, this behavior correlates with the degree of demyelination and the presence or absence of axonal damage. Specifically, pure demyelination suppresses the N1 component's amplitude, area, and half‐width of the CAP, indicating a reduction in the number of myelinated axons recruited to the CAP. The amplitude and area of the N2 component were enhanced after demyelination, as demyelinated axons would be recruited to the N2 component of the CAP. In response to WM damage that includes demyelination and axonal injury, as occurs in the L‐NIO WMS model, the amplitude and area of both the N1 and N2 components of the CAPs were suppressed. As myelination and axon diameter directly influence the conduction velocity, reduction in these measurements after WMS decreased the conduction velocity of myelinated axons (Figure 2). Thus, the N1 half‐width and velocity, in concert with the N1 and N2 amplitudes and areas, can be used to differentiate between pure demyelination and demyelination combined with axonal injury. The N1 half‐width was more sensitive to demyelination, whereas the conduction velocity was more sensitive to axonal injury.

## CONFLICT OF INTEREST

The authors declare that they have no competing financial interests to disclose.
