# Supplementary material for: Distinctive functional deficiencies in axonal conduction associated with two forms of cerebral white matter injury
Source: CNS Neurosci Ther. 2019 May 29;25(9):1018–29. doi: 10.1111/cns.13155 (PMC6698976; doi:10.1111/cns.13155)
Supplement: Supplementary file 1 [file CNS-25-1018-s001.docx]

**Supplementary Figure:**

**
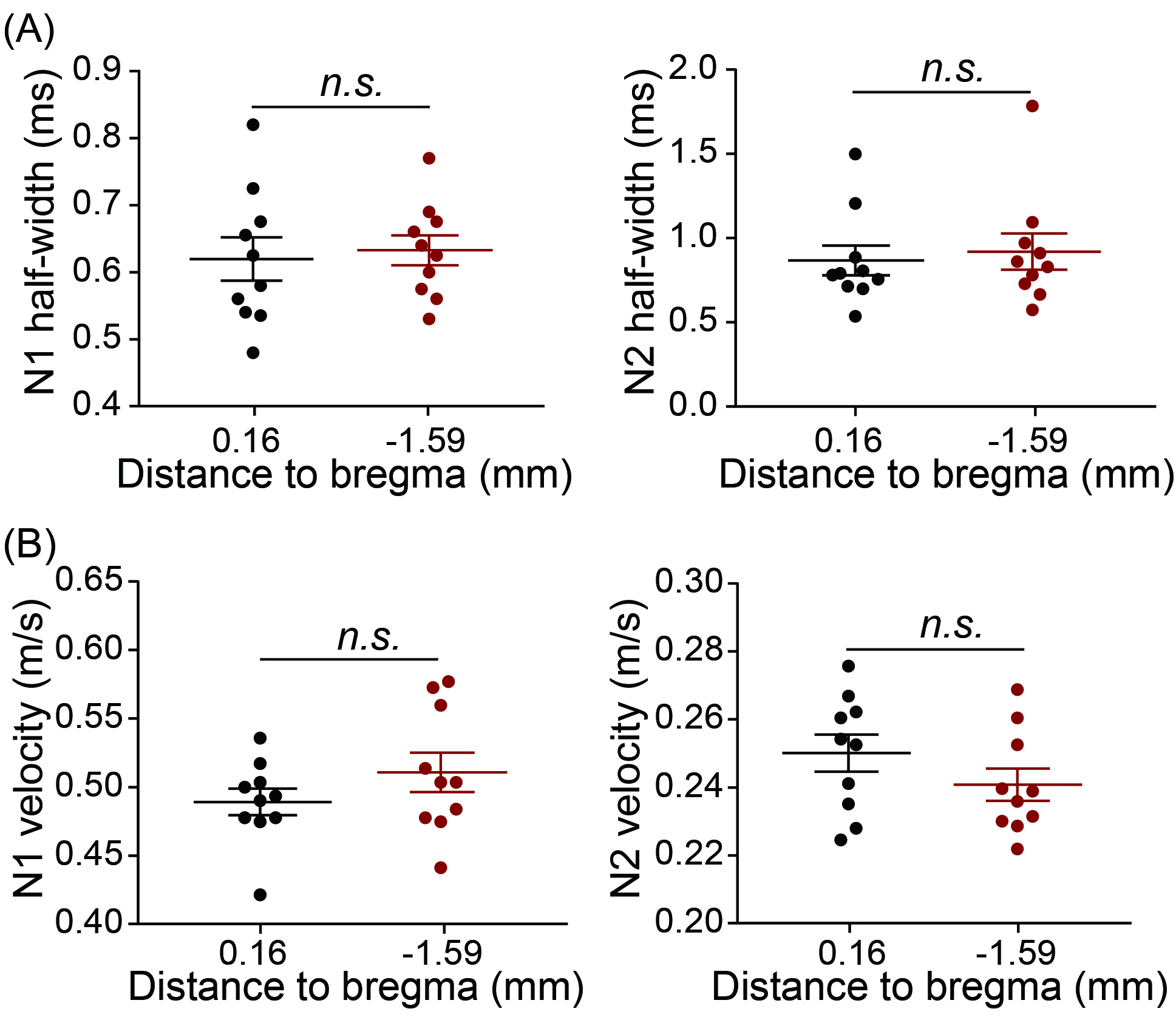
**

**Figure. S1**. There are no significant differences on half-width and axonal conduction velocity between brain sections at bregma 0.16mm and -1.59mm. (A) Comparison of N1 (left panel) and N2 (right panel) half-width between brain sections at bregma +0.16 mm and bregma -1.59 mm under different stimulus strength (0.25-2 mA). (B) Comparison of N1 (left panel) and N2 (right panel) conduction velocity between brain sections at bregma +0.16 mm and bregma -1.59 mm under different stimulus strength (0.25-2 mA). The results are presented as the mean ± SEM. n.s., statistically non-significant difference. n = 10 mice.


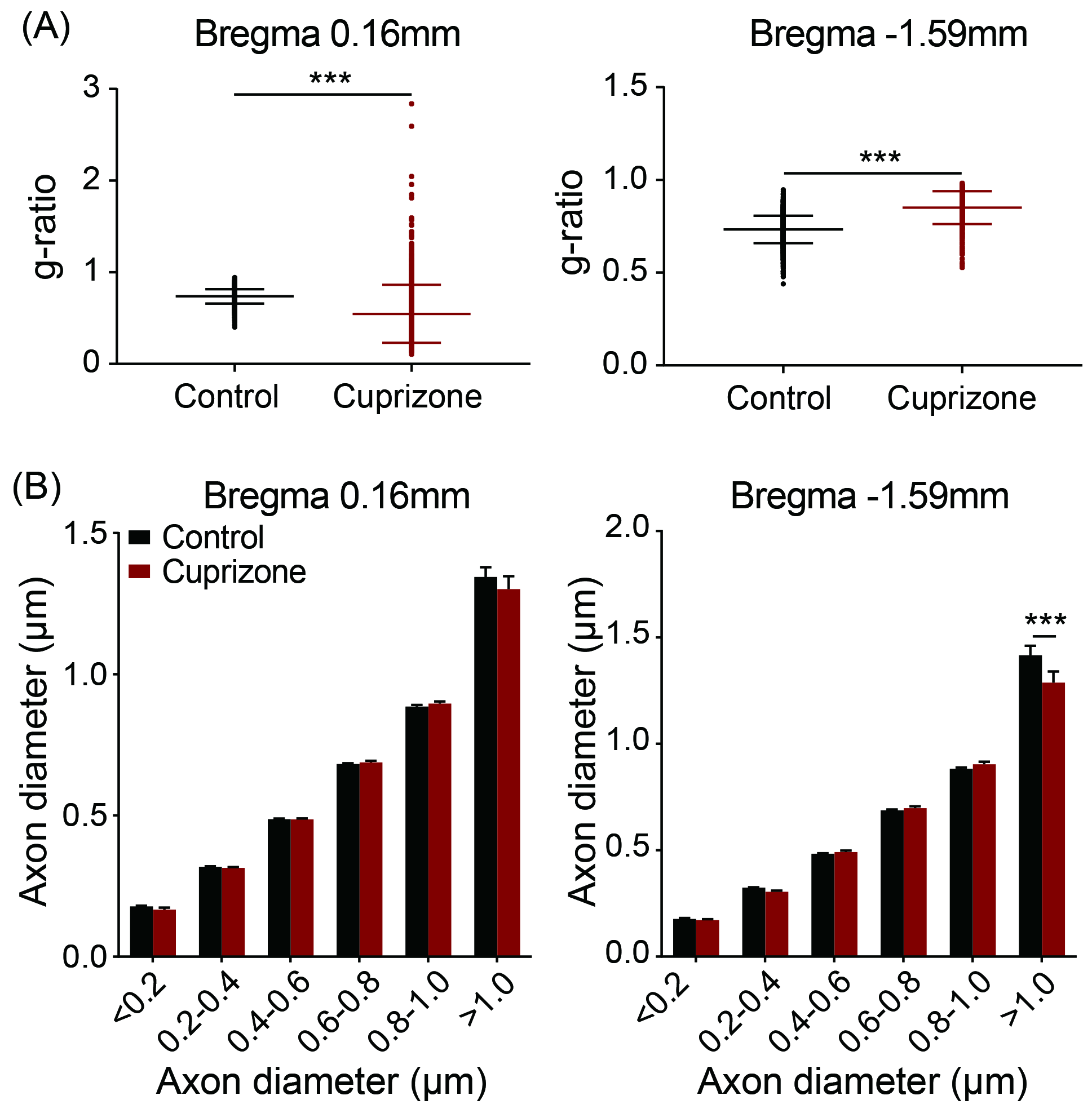


**Figure. S2**. Axon diameters are the same between normal and demyelinated mice across different locations. (A) Comparison of overall g-ratio on brain sections at bregma +0.16mm (left panel) and bregma -1.59mm (right panel) between normal and demyelinated mice. (B) Comparison of axon diameter based on different scales on brain sections at bregma +0.16mm (left panel) and bregma -1.59mm (right panel) between normal and demyelinated mice. The results are presented as the mean ± SEM. *** p < 0.001. n = 6-8 mice.

**Appendix S1. Supplementary Methods**

1. Induction of white matter stroke

Male C57BL/6J mice (8-10-week old) were initially exposed to 3% isoflurane in 67:30% N_2_O:O_2_ to induce anesthesia. Mice were placed in a stereotaxic apparatus where the anesthetic plane was maintained with 1.5% isoflurane in 67:30% N_2_O:O_2,_ at which time white matter stroke was induced as previously described.^1^ Briefly, a hole was drilled into the skull to expose the sensorimotor cortex next to the midline. A pulled glass micropipette affixed to the stereotaxic arm and connected to a pressure pump was used to deliver N5- (1-iminoethyl)-L-ornithine (L-NIO; 27 mg/mL in sterile physiological saline; EMD/Millipore, Burlington, MA, USA). The tip of the micropipette was inserted through the cortex into the underlying white matter at an angle of 36° with a dorsal-medial to ventrolateral injection path to minimize cortical damage. L-NIO was injected at 3 sites (150 nl each) at the following coordinates: AP +0.22, ML +0.22 mm, DV -2.1 mm; AP +0.7 mm, ML +0.15 mm, DV -2.16 mm; AP +1.21 mm, ML +0.15 mm, DV -2.18 mm. The sham mice were injected with equivalent saline. After the final injection, the micropipette was kept in place for an additional 5 min to prevent reflux, and was then slowly withdrawn.

2. CAP Measurements

CAPs in the CC were evaluated as previously described^2^. Mice were anesthetized with isoflurane (5%), decapitated, and then their brains were quickly removed. Coronal slices (350 µm) were cut on a vibratome and placed in artificial cerebrospinal fluid (aCSF; 119 mmol/L NaCl, 2.5 mmol/L KCl, 1 mmol/L NaH_2_PO_4_, 1.3 mmol/L MgSO_4_, 2.5 mmol/L CaCl_2_, 26.2 mmol/L NaHCO_3_, 10 mmol/L glucose; pH 7.4) that had been pre-gassed with a 95% O_2_/5% CO_2_ mixture. Sections were incubated in the aCSF for 0.5 hours at 34 °C, and then for 1 hour at room temperature (22 °C). Before recordings were made, the brain slices were transferred to a recording chamber where they were submerged in and perfused with aCSF (22 °C) at a constant rate of 4 to 5 mL/minute. Recordings were made at room temperature to differentiate the myelinated (N1) and unmyelinated (N2) components of the CAPs. A bipolar, tungsten stimulating electrode (inter-tip distance, 100 μm) was inserted into the CC approximately 100 μm deep and 1 mm lateral to the midline. A glass extracellular recording pipette (3 to 5 MΩ tip resistance when filled with aCSF) was lowered into the CC. For recordings, the distance between the stimulus electrode and the recording electrode was 0.75 mm or 1 mm. Data were acquired using Axon™ pClamp 10 software (Molecular Devices, San Jose, CA USA), sampled at 2–10 kHz, and filtered at 1 kHz. Off-line analysis was done with Clampfit 10 software (Molecular Devices, San Jose, CA USA). The N1 and N2 amplitudes were determined by measuring between the tip of the peak to the bottom of this wave (see the red line in Fig. 3A). The conduction velocity = conduction distance (0.75 mm) / conduction time (t1 or t2) (Fig. 3A).

3. Immunohistochemical staining

Brain sections derived from mice (8 weeks old) were blocked and incubated with the following primary antibodies: mouse anti-non-phosphorylated neurofilament H monoclonal (SMI-32; Covance, Princeton, NJ, USA), rabbit anti-myelin basic protein (MBP; Millipore, Billerica, MA, USA), and mouse anti-neurofilament 200 (NF200; Millipore, Billerica, MA, USA), for 1 hour at room temperature followed by overnight incubation at 4 °C. After washing, sections were incubated in a mixture of anti-rabbit secondary antibody conjugated with DyLight 488 (Jackson ImmunoResearch Laboratories, West Grove, PA, USA) and anti-mouse secondary antibody conjugated with DyLight 594 (Jackson ImmunoResearch Laboratories, West Grove, PA, USA) for 1 hour at room temperature. Sections were then counterstained with 4’,6-diamidino-2-phenylindole (Thermo Scientific, Pittsburgh, PA, USA) for 2 minutes at room temperature, followed by mounting with Fluoromount-G (Southern Biotech, Birmingham, AL, USA). For densitometric analysis, a computerized camera-based NIH Image analysis system (available at http://rsb.info.nih.gov/nih-image/) was used.^2^ Briefly, areas of interest in the damaged peripheral zone were digitally captured as TIFF images at equivalent exposure time. The images were then binarized and segmented under a consistent threshold (50%). Next, the total black pixels per image were counted.

4. Electron microscopic studies

Electron microscopic analysis was used to assess axonal morphology/damage in the CC, as described previously^2^. Briefly, eight week-old mice were deeply anesthetized with isoflurane (5%). Subsequently, they were decapitated and had their brains quickly removed. Coronal slices (350 µm) were cut on a vibratome and fixed in a solution containing 2.5% glutaraldehyde for 24 h. These callosal slices matched those slices used for electrophysiological assessment. All sections were osmicated, stained in uranyl acetate, dehydrated, and embedded in Medcast Resin (Ted Pella Inc., Redding, CA, USA), using the flat mounting technique with plastic slides and coverslips. Regions of interest within the CC were then identified, cut out, mounted on plastic studs, and serially sectioned with a diamond knife. The resulting thin sections were collected on formvar-coated slotted grids and stained with lead citrate. Using a Philips CM120 electron microscope (Royal Dutch Philips Electronics Ltd., Amsterdam, Holland, The Netherlands), sections were screened and representative images acquired on film at 20000x. Fibers were assessed for evidence of any structural/subcellular perturbation, such as degradation of the myelin sheath, ultrathin myelin sheath, and morphologic changes in neurofilaments.

**References**

1. Nunez S, Doroudchi MM, Gleichman AJ, et al. A Versatile Murine Model of Subcortical White Matter Stroke for the Study of Axonal Degeneration and White Matter Neurobiology. *Jove-J Vis Exp.* 2016(109).

2. Wang GH, Shi YJ, Jiang XY, et al. HDAC inhibition prevents white matter injury by modulating microglia/macrophage polarization through the GSK3 beta/PTEN/Akt axis. *P Natl Acad Sci USA.* 2015;112(9):2853-2858.
